# OP24 Cost Effectiveness Of Monitoring Ocular Hypertension Based On A Risk Prediction Tool

**DOI:** 10.1017/S0266462325100822

**Published:** 2025-12-29

**Authors:** Hangjian Wu, Gus Gazzard, Anthony King, James Morgan, David Wright, David Crabb, Yemisi Takwoingi, Augusto Azuara-Blanco, Verity Watson, Rodolfo Hernández

## Abstract

**Introduction:**

Glaucoma is a leading cause of blindness. Ocular hypertension (OHT) is a key risk factor modifiable by regular monitoring and treatment. Population aging leads to rapid rise of OHT patients, and thus overburdens the UK healthcare system, but innovative technology may provide a solution. We assessed the cost effectiveness of making treatment decisions based on a validated risk prediction (RP) tool.

**Methods:**

A discrete event simulation model was constructed to compare the cost effectiveness of an alternative care pathway in which the treatment decision was guided by a validated RP tool in secondary care against decision-making based on standard care (SC). Individual patient sampling was used. Patients diagnosed with OHT and with an intraocular pressure of at least 24 mmHg entered the model with a set of predefined individual characteristics related to their risk of conversion to glaucoma. These characteristics were retrieved from electronic medical records (n=5,740). Different stages of glaucoma were modeled following conversion to glaucoma.

**Results:**

Almost all (99%) patients were treated using the RP strategy, and less than half (47%) of the patients were treated using the SC strategy. The RP strategy produced higher cost but also higher quality-adjusted life years (QALYs) than the SC strategy. The RP strategy was cost effective compared with the SC strategy in the base case analysis, with an incremental cost-effectiveness ratio value of GBP11,522 (USD15,843). The RP strategy had a 96 percent probability of being cost effective under a GBP20,000 (USD27,500) per QALY threshold.

**Conclusions:**

The use of an RP tool for the management of patients with OHT is likely to be cost effective. However, the generalizability of the result might be limited due to the high risk nature of this cohort and the specific RP threshold used in the study.

